# A tumour-spheroid manufacturing and cryopreservation process that yields a highly reproducible product ready for direct use in drug screening assays

**DOI:** 10.1098/rsif.2023.0468

**Published:** 2023-10-11

**Authors:** Md. Shafiullah Shajib, Kathryn Futrega, Anthony M. Davies, Rose Ann G. Franco, Eamonn McKenna, Bianca Guillesser, Travis J. Klein, Ross W. Crawford, Michael R. Doran

**Affiliations:** ^1^ School of Biomedical Science, Faculty of Health, Queensland University of Technology (QUT), Brisbane, Queensland, Australia; ^2^ Centre for Biomedical Technologies, School of Mechanical, Medical, and Process Engineering, Faculty of Engineering, Queensland University of Technology (QUT), Brisbane, Queensland, Australia; ^3^ Translational Research Institute, Brisbane, Queensland, Australia; ^4^ Department of Health and Human Services, National Institute of Dental and Craniofacial Research (NIDCR), National Institutes of Health (NIH), Bethesda, MD, USA; ^5^ Vale Life Sciences, Brisbane, Australia

**Keywords:** tumour-spheroids, microwell, cryopreservation, buoyancy, metabolic activity

## Abstract

If it were possible to purchase tumour-spheroids as a standardised product, ready for direct use in assays, this may contribute to greater research reproducibility, potentially reducing costs and accelerating outcomes. Herein, we describe a workflow where uniformly sized cancer tumour-spheroids are mass-produced using microwell culture, cryopreserved with high viability, and then cultured in neutral buoyancy media for drug testing. C4-2B prostate cancer or MCF-7 breast cancer cells amalgamated into uniform tumour-spheroids after 48 h of culture. Tumour-spheroids formed from 100 cells each tolerated the cryopreservation process marginally better than tumour-spheroids formed from 200 or 400 cells. Post-thaw, tumour-spheroid metabolic activity was significantly reduced, suggesting mitochondrial damage. Metabolic function was rescued by thawing the tumour-spheroids into medium supplemented with 10 µM *N*-Acetyl-l-cysteine (NAC). Following thaw, the neutral buoyancy media, Happy Cell ASM, was used to maintain tumour-spheroids as discrete tissues during drug testing. Fresh and cryopreserved C4-2B or MCF-7 tumour-spheroids responded similarly to titrations of Docetaxel. This protocol will contribute to a future where tumour-spheroids may be available for purchase as reliable and reproducible products, allowing laboratories to efficiently replicate and build on published research, in many cases, making tumour-spheroids simply another cell culture reagent.

## 1. Introduction

Organoids, microtissues or tumour-spheroids are terms used to describe small clusters of cells cultured *in vitro* that often take on tissue- or organ-like features. These cellular amalgamations can be useful in promoting stem cell differentiation down various lineages [1,2], including into multiple lineages yielding complex organ-like features, and in encouraging cells to behave in a more physiological manner [1–3], including, for example, how they might metabolise or respond to drugs [3]. Because of these characteristics, many researchers now argue that tissue culture should evolve to focus on 3D cultures rather than 2D cultures, which some view as antiquated [4,5]. These researchers are in lockstep with the field, as a PubMed search for ‘organoid' or ‘microtissue' or ‘tumour-spheroid' yielded 9013 publications in 2012 versus 21 678 publications in 2022, an approximately 2.4-fold increase over a decade and a trend that appears to be increasing exponentially. Correspondingly, the organoid market is anticipated to experience 21% compound annual growth, yielding a $3.4 billion market by 2027 [6]. This paper focuses on amalgamated cancer cells, and thus when referring to our own experimental data we will describe the cell clusters as tumour-spheroids.

While 3D tumour-spheroids may offer better tools to interrogate biology *in vitro*, their incremental complexity over traditional 2D monolayers may create greater challenges for data reproducibility, potentially contributing to the so-called reproducibility crisis [7]. The reproducibility crisis refers to the observation that many scientific studies are difficult or impossible to replicate or reproduce, including many studies reported in high-impact journals [7,8]. Freedman *et al*. conducted an economic analysis where they estimated that irreproducible preclinical research costs the USA $28 billion annually [9]. Tumour-spheroids, or their organoid or microtissue counterparts, are increasingly viewed as critical tools to better inform preclinical studies and, in some cases, are anticipated to be used in place of preclinical animal models. The rapid advancement of biomedical research has been facilitated by the widespread commercial availability and use of standardised reagents such as recombinant proteins, including growth factors and antibodies, pre-made cell culture medium, cell lines and even animal models. It is possible that a necessary step in the continued growth in tumour-spheroids use is the ability for laboratories to purchase standardised tumour-spheroid products, thus reducing laboratory-to-laboratory variability and enabling precise replication of published studies that used commercial tumour-spheroids, or their organoid or microtissue counterparts.

STEMCELL Technologies already sells cryopreserved mouse intestinal, hepatic or pancreatic organoids [10]. STEMCELL Technologies recommends that organoids be thawed and cultured for a few passages before use in experimental studies [11–14]. While these organoids are not ready for direct use in experiments, they do offer a standardised product and medium formulation kit, which is moving the field in a necessary direction. A major application for tumour-spheroids is in cancer research, particularly in drug testing [15–17]. We reasoned that a logical evolution of the use of tumour-spheroids would be their standardisation as a cryopreserved product that could be used directly following thaw in drug testing assays. For such a process to be feasible, the tumour-spheroid product would need to maintain high viability following cryopreservation, be easily manipulated in large-scale assays, and finally, the fresh and cryopreserved tumour-spheroid product would have to behave similarly in drug assays. Based on these objectives, we considered currently available tumour-spheroid technologies and designed experiments to optimise the process to meet the above criteria.

Many different strategies are used to assemble cells into organoids, microtissues or tumour-spheroids, including non-adherent culture surfaces [18], microfluidics [19], hanging drops [20], gels [21] and microwell platforms [15–17]. We used a microwell platform in this study, as this enables the efficient generation of thousands of uniformly sized tumour-spheroids, which are ideal inputs into a complex bioprocess. Cryopreservation requires the harvest of the tumour-spheroids from microwells, after which there is potential for tumour-spheroids to settle in tubes and aggregate. Aggregation of tumour-spheroids results in the loss of the uniform product that made them a valuable process input, and it complicates their consistent distribution into well plates. To facilitate fluidic handling and to prevent undesirable aggregation, we froze and cultured thawed tumour-spheroids in neutral buoyancy medium. Cryopreservation was performed in CryoStor CS10 (STEMCELL Technologies) and culture was performed in Happy Cell ASM (Vale Life Sciences, Australia) 3D culture medium; in both media formulations tumour-spheroids are at least partially buoyant.

To evaluate the above approach, we mass-manufactured uniform tumour-spheroids from C4-2B prostate cancer or MCF-7 breast cancer cells using in-house fabricated microwells. We optimised a freeze–thaw protocol using CryoStor CS10 and mitigated reactive oxygen species (ROS)-induced damage in thawed tumour-spheroid cultures by supplementing the medium with *N*-Acetyl-l-cysteine (NAC), a ROS scavenger [22]. The tumour-spheroids were revitalised in Happy Cell ASM 3D culture media supplemented with NAC. Cryopreserved and non-cryopreserved tumour-spheroids were treated with an anti-cancer drug and characterised for viability, DNA content and metabolic activity.

## 2. Material and methods

### 2.1. Cell lines and cell expansion

The prostate cancer cell line, C4-2B [23] and breast cancer cell line, MCF-7 [24], were used for this study. The cells were expanded in T175 flasks using high glucose Dulbecco's modified Eagle's medium (HG-DMEM) with pyruvate and GlutaMAX, supplemented with 100 U/ml penicillin/streptomycin (Pen/Strep; Gibco) and 10% FBS (Gibco). Cell cultures were maintained in a humidified incubator at 5% CO_2_ and 37°C. Cultures were passaged at approximately 80% confluency using 0.25% Trypsin/EDTA (v/v) (Thermo Fisher). Cells were re-seeded at 1500 cells/cm^2^ in fresh flasks and expanded as required for tumour-spheroid manufacture or further experimentation.

### 2.2. Microwell fabrication and preparation

Tumour-spheroids were assembled in microwells, using microwell plates fabricated in-house, as previously described [25,26]. Our microwell platform design is based on a concept originally described by Dr Peter Zandstra's laboratory [27]. Briefly, sheets of polydimethylsiloxane (PDMS, SYLGARDTM 184 Silicone Elastomer Kit, Dow Silicones Corporation, Midland, MI, USA) were cast on a polystyrene mould, that had the inverse shape of the microwell pattern. Microwell dimensions were 360 × 360 µm wide by 180 µm deep, yielding a patterned surface with 600 microwells/cm^2^. A wad punch (Amazon.com) was used to punch 1.6 cm diameter discs from the PDMS sheets. Using silicone glue, disc inserts were anchored in 24-well plates. Each disc insert had approximately 1200 microwells and was thus capable of generating approximately 1200 tumour-spheroids. To sterilise plates, 1 ml of 80% (v/v) ethanol was added to the well, and the plate was centrifuged at 3000×*g* to displace bubbles from the microwells and under the inserts. Following centrifugation, the plates were submerged entirely in an 80% ethanol bath for 1 h at room temperature. The microwells were washed thrice with sterile deionised water (Thermo Fisher Scientific), dried overnight at 60°C in an oven, and stored at room temperature until needed. Prior to cell seeding, microwell surfaces were treated with sterile 5% Pluronic F-127 Sigma-Aldrich) in Dulbecco's phosphate buffered saline (DPBS) (w/v). Pluronic solution (1 ml) was added to each well, and the plate was centrifuged at 3000×*g* for 10 min to displace air bubbles from microwells. Pluronic F-127 absorbs and coats the PDMS surface, thereby reducing protein adsorption and cell attachment onto the PDMS surface, which promotes cell aggregation [27,28]. Excess Pluronic F-127 was rinsed out thrice using DPBS.

### 2.3. Tumour-spheroid manufacture

As previously described [16], C4-2B or MCF-7 tumour-spheroids were assembled using the microwell platform from single-cell suspensions. Initial screening evaluated tumour-spheroids of 100, 200 or 400 cells each. This was achieved by suspending C4-2B or MCF-7 at 1.2 × 10^5^ cells, 2.4 × 10^5^ cells or 4.8 × 10^5^ cells in 1 ml of HG-DMEM supplemented with 10% FBS and 100 U/ml Pen/Strep. Cells were pelleted into microwells by centrifuging the plate at 300×*g* for 5 min. The distribution of cells into the microwells and their subsequent aggregation was confirmed using an Olympus CKX14 microscope, digital camera (Olympus DP26, Japan) and imaging software (CKX14, CellSens Entry). Prior to cryopreservation, tumour-spheroids were cultured for 48 h in a 5% CO_2_ atmosphere at 37°C.

### 2.4. Cryopreservation and thawing of tumour-spheroids

The C4-2B and MCF-7 tumour-spheroids were gently and aseptically aspirated from microwells. Approximately 1000 tumour-spheroids per millilitre of cryoprotectant were loaded into 1.2 ml internal threaded polypropylene Cryo.s freezing tubes (Greiner Bio-One, Austria). We assessed C4-2B tumour-spheroid cryopreservation in standard laboratory cryopreservation solution, 90% FBS supplemented with 10% DMSO, or alternatively in the commercial cryoprotectant Cryostor CS10. The manufacturer's instructions were followed when using CS10. Briefly, the culture media was removed from wells and the tumour-spheroids were resuspended in cold (4°C) CS10 solution. The suspended tumour-spheroids were incubated at 4°C for 10 min. The CS10 or FBS plus DMSO suspended tumour-spheroids were transferred to a controlled cooling rate container (Mr. Frosty; Thermo Fisher Scientific) and frozen at −80°C for 24 h. The tumour-spheroid-containing vials were transferred to liquid nitrogen for extended storage periods. The cryopreserved tumour-spheroids were rapidly thawed at 37°C in a water bath, gently swirling the submerged cryotube base. The cryomedia was diluted 1 : 10 with HG-DMEM supplemented with 1% PenStrep, 10% FBS and 10 µM NAC. After observing that C4-2B or MCF-7 tumour-spheroids amalgamated in the HG-DMEM culture media, compromising downstream cultures, we subsequently cultured tumour-spheroids in medium supplemented with Happy Cell ASM medium (Vale Life Sciences, Australia) and 10% FBS and Pen/Strep. Tumour-spheroids are semi buoyant in Happy Cell ASM medium, and this prevents their aggregation. In the optimised protocol, tumour-spheroids were cryopreserved in CS10, then revived in 1 ml of medium formulated from HG-DMEM, supplemented with Happy Cell ASM, 10% FBS and Pen/Strep and 10 µm NAC. Revival cultures were performed in 5 ml flat base screw cap tubes (Sarstedt, Australia), leaving the screw cap loose in a humidified 5% CO_2_ and 37°C incubator for 24 h. Following this 24-h revival culture, the Happy Cell ASM suspension was inactivated using the Inactivation Solution that is provided with the product. This process results in the tumour-spheroids no longer being buoyant in the Happy Cell ASM, sinking to the bottom of vessel and allows for full media exchange. The tumour-spheroids were washed with HG-DMEM and then approximately 50 tumour-spheroids were resuspended in 200 µl of fresh HG-DMEM supplemented with Happy Cell ASM, 10% FBS and Pen/Strep in each well of ultra-low binding 96-well plates (Corning 3474 Costar). Images of the tumour-spheroids were captured using an Olympus CKX14 microscope. Tumour-speroid diameters were measured using a digital camera (Olympus DP26, Japan) and imaging software (CKX14, CellSens Entry) before suspending into Happy Cell ASM media. These data were compared with the non-cryopreserved tumour-spheroids cultured over the same time period.

### 2.5. Determination of the metabolic activity of cryopreserved tumour-spheroids

The metabolic activity of post-thawed tumour-spheroids was assessed using AlamarBlue solution containing Resazurin (Thermo Fisher Scientific). Resazurin is a blue-violet dye that is reduced by NADH or NADPH reductase enzymes, turning pink or into Resorufin, which has a strong fluorescence emission [29,30]. The emitted fluorescence signal is used as a measure of metabolic activity and, in many studies, as an indirect measure of cell viability. The AlamarBlue assay was conducted following the procedure described previously [17]. Briefly, tumour-spheroid cultures were supplemented with 3% AlamarBlue solution and incubated in a 5% CO_2_, 37°C incubator. AlamarBlue fluorescence was recorded at 544 nm excitation and 590 nm emission from the bottom of the plate using a CLARIOstar Plus microplate reader (BMG LABTECH) every hour for 4 h. A titration of C4-2B or MCF-7 cells was characterised in parallel, providing a viable cell standard curve for the AlamarBlue assays.

### 2.6. Tumour-spheroid viability assay

The viability of tumour-spheroids was assessed using LIVE/DEAD cell viability solution (Thermo Fisher Scientific), following manufacturer's instructions. Post-thaw recovered or drug-treated tumour-spheroids were washed with PBS following the AlamarBlue assay. As a negative control, tumour-spheroids were devitalised in 80% ethanol for 30 min, yielding a dead cell control. Approximately 10 tumour-spheroids were distributed into wells in a black 96-well plate and stained with 2 µM calcein-AM (Green, stains live cells) and 4 µM of ethidium homodimer-1 (Red, stains dead cells). The tumour-spheroids immersed in the staining solution were incubated in the dark for 45 min at room temperature. Tumour-spheroids were washed with PBS to remove excess stain and then assessed using an Olympus FV3000 confocal microscope.

### 2.7. DNA quantification of cryopreserved tumour-spheroids

The DNA content of tumour-spheroids was quantified as secondary means to estimate viability. The DNA quantification was performed using the Quant-iT PicoGreen dsDNA Assay Kit (Thermo Fisher Scientific) as per the manufacturer's instructions. Briefly, tumour-spheroids were lysed using a combination of 1% Tween 20 in TE buffer (20 mM Tris–HCl, 2 mM EDTA, pH 7.5) and two freeze/thaw cycles, as described previously [17]. Twenty-five microlitres of cell lysate was mixed with an equal volume of PicoGreen solution and transferred into half-volume black 96-well plates (Corning Costar). Fluorescence was read from the top of the plate at 485 nm excitation and 520 nm emission using a CLARIOstar Plus microplate reader (BMG LABTECH). A titration of lambda-DNA was used to generate a standard curve.

### 2.8. Drug response assay of cryopreserved tumour-spheroids

The drug response of cryopreserved tumour-spheroids was performed using a cytotoxic drug, Docetaxel [31]. After post-thaw treatment and 24 h of recovery, tumour-spheroids were distributed into ultra-low binding 96-well plates (Corning Costar). Approximately 50 tumour-spheroids were suspended in 200 µl of Happy Cell ASM media supplemented with 10% FBS. Tumour-spheroids were treated with Docetaxel at concentrations ranging from 0 to 100 nM, prepared in DMSO (1.5% v/v final). The cryopreserved and non-cryopreserved tumour-spheroids were incubated in a 5% CO_2_ incubator at 37°C following drug treatment. After 72 h, the culture media was supplemented with 3% AlamarBlue to determine the metabolic output of the different cultures in response to the drug. Fluorescence was read at 544 nm excitation and 590 nm emission using a CLARIOstar Plus microplate reader (BMG LABTECH), 4 h following the addition of AlamarBlue. Parallel drug assays were performed with C4-2B or MCF-7 cells cultured in monolayers in standard tissue culture-treated 96-well plates (Corning Costar). Each well was seeded with 5000 cells in 200 µl HG-DMEM supplemented with 10% FBS and 100 U/ml pen/step. Cultures were maintained in a 5% CO_2_ and 37°C incubator for 24 h before initiating Docetaxel treatment. Like tumour-spheroids, the monolayer cells were incubated with the drug for 72 h before the addition of AlamarBlue to the medium. During the metabolic AlamarBlue assays, a titration of the cancer cells was performed in parallel to function as a standard curve. The quantity of DNA in both the drug-treated cryopreserved and non-cryopreserved tumour-spheroids, as well as in the monolayer cultures, was assessed at the end of the metabolic assays to validate any gain or loss in cell number.

### 2.9. Statistical analysis

Data are presented as mean ± s.d. for four biological replicates (*n* = 4). Graph generation and statistical analysis were performed using GraphPad Prism version 8 software. The significance of the difference between the groups was calculated by one-way analysis of variance (ANOVA) following Tukey's post-hoc test. A *p*-value less than 0.05 was considered statistically significant.

## 3. Results

### 3.1. Tumour-spheroid culture and size optimisation

**Figure 1.**
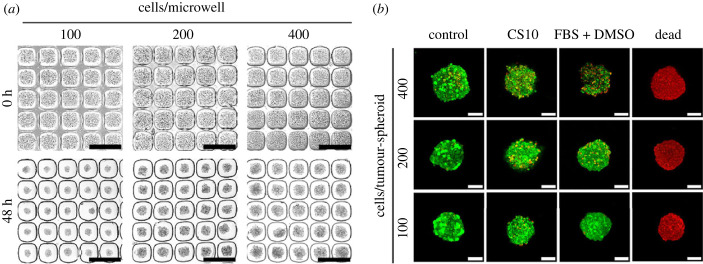
(*a*) C4-2B cells were seeded at approximately 100, 200 or 400 cells per well. The top row shows the different densities of cells immediately after seeding into microwells. The bottom row shows tumour-spheroid formation following 48 h of culture. The smaller-sized tumour-spheroids resulted in greater uniformity. Images scale bar = 500 µm. (*b*) Tumour-spheroids manufactured from 100, 200 or 400 cells and cryopreserved in 90% FBS + 10% DMSO or Cryostor CS10. Tumour-spheroids were thawed and permitted to recover in HG-DMEM media supplemented with 10% FBS and 1% Pen/Strep in a 5% CO_2_, 37°C incubator. Tumour-spheroids were stained with LIVE/DEAD dye and imaged uning confocal microscopy. The left column (control) shows non-cryopreserved tumour-spheroids. The right column (red coloured tumour-spheroids) shows dead control tumour-spheroids prepared in 80% ethanol for 30 min and stained with ethidium homodimer-1. Red and green fluorescence represent dead and live cells in the tumour-spheroids, respectively. Image scale bar = 50 µm.

In preliminary experiments, we aimed to establish an ideal tumour-spheroid size and cryopreservation medium for process optimisation. Tumour-spheroids were formed from 100, 200 or 400 C4-2B or MCF-7, and then cultured for 48 h and characterised using light microscopy. As depicted in figure 1*a*, C4-2B prostate cancer and MCF-7 breast cancer cells aggregated in microwells, yielding *relatively* uniform size and shape following 48 h of culture. Uniformity of spherical shape and size was greatest for tumour-spheroids assembled from approximately 100 cells each (figure 1*a*). While the size and shape of the tumour-spheroids was not perfectly uniform, the microwell platform offers relatively good control over these features and high efficiency.

Next, we compared cryopreservation outcomes for tumour-spheroids generated from 100, 200 or 400 C4-2B cells preserved in either 90% FBS + 10% DMSO or in Cryostor CS10. Following thaw, tumour-spheroids were assessed using Thermo Fisher's LIVE/DEAD cell viability kit (figure 1*b*). Relative cell viability was greatest for 100 cell tumour-spheroids cryopreserved in Cryostor CS10, and the Cryostor CS10 cryopreservation medium is advantageous in this application as tumour-spheroids are slightly buoyant in this medium which results in reduced settling and tumour-spheroid aggregation. In subsequent studies, tumour-spheroids were assembled from 100 cells each and cryopreserved in Cryostor CS10. The diameter of tumour-spheroids generated from 100 C4-2B or 100 MCF-7 cells was assessed before and after cryopreservation. C4-2B tumour-spheroids (123.9 ± 14.3 µm) had a significantly greater diameter than MCF-7 tumour-spheroids (99.9 ± 20.0 µm), but in both cases diameters were similar before and after cryopreservation (electronic supplementary material, figure S1).

### 3.2. Using neutral buoyancy to maintain discrete tumour-spheroids

**Figure 2.**
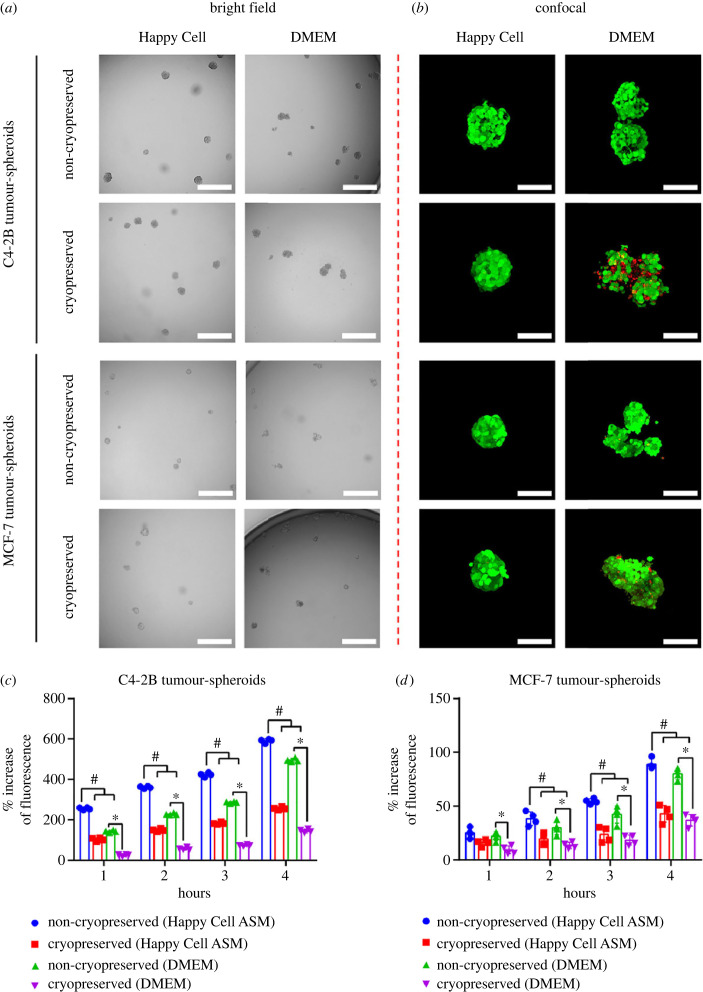
(*a*,*b*) Morphology and viability of cryopreserved and non-cryopreserved tumour-spheroids. The cryopreserved tumour-spheroids were revived for 24 h in HG-DMEM supplemented with 10% FBS plus 1% Pen/Strep or HG-DMEM supplemented Happy Cell ASM, 10% FBS and 1% Pen/Strep in a 5% CO_2_, 37°C incubator. (*a*) Bright-field images show morphology of C4-2B and MCF-7 tumour-spheroids. Images scale bar 500 µm. (*b*) LIVE/DEAD stained tumour-spheroids imaged using a confocal microscope. Red and green fluorescence represent dead and live cells, respectively. Image scale bars = 50 µm. (*c*,*d*) AlamarBlue metabolic signal from cryopreserved tumour-spheroids. Cryopreserved tumour-spheroids were cultured in HG-DMEM or Happy Cell ASM media for 24 h after thaw. After the revival period, the culture media was supplemented with 3% AlamarBlue. The fluorescence conversion of AlamarBlue by (*c*) C4-2B tumour-spheroids and (*d*) MCF-7 tumour-spheroids was recorded for 4 h. The percentage (%) fluorescence signal was calculated relative to the control medium that contained no tumour-spheroids. Data are represented as the mean ± s.d. value of four replicates. * and # represent statistical significance (*p* < 0.05) compared to non-cryopreserved tumour-spheroids cultured in Happy Cell ASM or HG-DMEM, respectively.

While tumour-spheroids of 100 cells each cryopreserved in CS10 yielded high cell viability, the tumour-spheroids had a propensity to aggregate with each other in downstream cultures. Tumour-spheroid aggregation is problematic, as this uncontrolled aggregation process yields variably sized tumour-spheroids, which have different surface area to volume ratios and establish different diffusion gradients. These characteristics can influence cell biology as well as how tumour-spheroids respond in drug assays [32–34]. Additionally, aggregation prevents uniform distribution of tumour-spheroids into well plates, which ultimately make them unusable in quantitative assays. To mitigate tumour-spheroid aggregation following cryopreservation, we evaluated culturing thawed tumour-spheroids in neutral buoyancy Happy Cell ASM media. In neutral buoyancy Happy Cell ASM medium, tumour-spheroids rarely come into contact with each other, and thus do not aggregate. We directly compared fresh or thawed tumour-spheroids, that were cultured for 24 h in HG-DMEM supplemented with 10% FBS plus 1% Pen/Strep or HG-DMEM supplemented with Happy cell ASM, 10% FBS and 1% Pen/Strep (figure 2). For both fresh and thawed tumour-spheroids, aggregation was less frequent when the culture medium was supplemented with Happy Cell ASM (figure 2*a*). We assessed the viability of tumour-spheroids cultured in HG-DMEM or HG-DMEM supplemented with Happy Cell ASM, and found that neutral buoyancy resulted in greater cell viability (figure 2*b*). We speculate that when tumour-spheroids aggregate that this can make the cells more vulnerable to shear stresses induced during culture manipulation and that, for this reason, there is a viability benefit realised when culturing in neutral buoyancy medium.

To better quantify how the addition of Happy Cell ASM to the culture system might influence cell viability, we assessed tumour-spheroid metabolic activity of fresh or thawed tumour-spheroids cultured in HG-DMEM supplemented with 10% FBS plus 1% Pen/Strep or HG-DMEM supplemented with Happy cell ASM, 10% FBS and 1% Pen/Strep. Figure 2*c*,*d* shows that the conversion of AlamarBlue fluorescence was greater for non-cryopreserved C4-2B and MCF-7 tumour-spheroids, respectively. Tumour-spheroids cultured in Happy Cell ASM media outperformed HG-DMEM controls, but remained metabolically less active than non-cryopreserved controls.

### 3.3. NAC rescues metabolic activity following cryopreservation

Previous literature suggests that NAC protects mitochondria from damage following cryopreservation [35]. To assess if NAC could rescue mitochondrial function in thawed tumour-spheroids, we supplemented the culture medium with 0–20 mM NAC for the first 24 h of culture following cryopreservation. Based on the LIVE/DEAD microscopic assay, it appeared that NAC could be supplemented up to 10 mM without causing cell death in tumour-spheroids (electronic supplementary material, figure S2). We similarly performed titrations of NAC and assessed how this impacted AlamarBlue metabolic signals in fresh and cryopreserved tumour-spheroids (electronic supplementary material, figure S3). Cryopreserved tumour-spheroids revived in medium supplemented with 10 µM NAC yielded metabolic activity similar to non-cryopreserved control tumour-spheroids. At high concentrations of NAC (20 mM), which were observed to kill cells (electronic supplementary material, figure S2), the medium turned from pink to yellow, indicating a significant pH change and likely loss of medium buffering capacity (electronic supplementary material, figure S4). Collectively, based on these results, subsequent cryo-recovery experiments were performed using a 24 h recovery culture that was performed in HG-DMEM supplemented Happy Cell ASM, 10% FBS, 1% Pen/Strep plus 10 µM NAC.

**Figure 3.**
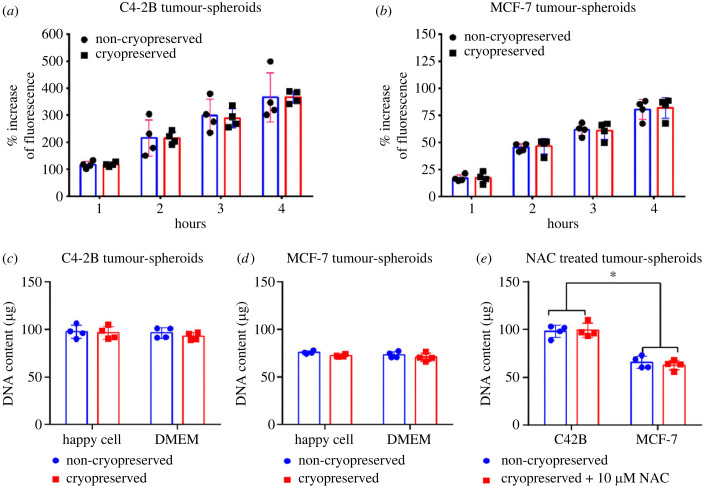
Metabolic conversion of cryopreserved tumour-spheroids treated with ROS inhibitor, *N*-Acetyl-l-cysteine (NAC). NAC was added at a concentration of 10 µM during thawing and during the 24 h of recovery time. Following this recovery period, tumour-spheroids were washed and suspended in Happy Cell ASM media. The media was supplemented with 3% AlamarBlue and fluorescence emission was tracked for (*a*) C4-2B tumour-spheroids and (*b*) MCF-7 tumour-spheroids over 4 h and compared with non-cryopreserved tumour-spheroids. The percentage (%) increase in fluorescence signal was calculated as relative to corresponding media-only controls that contained no tumour-spheroids. (*c*) C4-2B tumour-spheroids and (*d*) MCF-7 tumour-spheroids were recovered in HG-DMEM and Happy Cell ASM media and assessed for DNA quantity. (*e*) DNA content of cryopreserved tumour-spheroids treated with NAC (10 µM) during the recovery period in Happy Cell ASM media. Non-cryopreserved tumour-spheroids did not receive any treatment. Data are represented as the mean ± s.d. (*n* = 4). *p* < 0.05 was considered statistically significant.

Using this optimised recovery medium formulation, we assessed tumour-spheroid metabolic activity using the AlamarBlue assay as well as tumour-spheroid DNA content with or without cryopreservation (figure 3). When C4-2B (figure 3*a*) or MCF-7 (figure 3*b*) tumour-spheroids were permitted to recover in medium supplemented with 10 µM NAC, their metabolic activity was similar to non-cryopreserved tumour-spheroids. The DNA content in fresh and cryopreserved tumour-spheroids was measured using the PicoGreen assay. The C4-2B cryopreserved tumour-spheroids recovered in either HG-DMEM or Happy Cell ASM media had a similar quantity of DNA content compared to non-cryopreserved tumour-spheroids (figure 3*c*). The DNA content in cryopreserved MCF-7 tumour-spheroids was slightly reduced in Happy Cell ASM (72.2 ± 1.2 µg) and HG-DMEM (70.0 ± 4.2 µg) compared to non-cryopreserved Happy Cell ASM (75.6 ± 1.5 µg) and HG-DMEM (73.1 ± 3.2 µg) cultured tumour-spheroids, respectively (figure 3*d*). These differences were not statistically significant. NAC treatment during the thaw and recovery period did not alter the DNA content of the tumour-spheroids (figure 3*e*). These data suggest that cell number is not changing in tumour-spheroids during the cryopreservation process, and that manipulation with NAC is modifying respiration rather than tumour-spheroid cell content. The DNA content of C4-2B cryopreserved and non-cryopreserved tumour-spheroids were significantly greater than MCF-7 tumour-spheroids (figure 3*e*).

### 3.4. Tumour-spheroid drug assays

**Figure 4.**
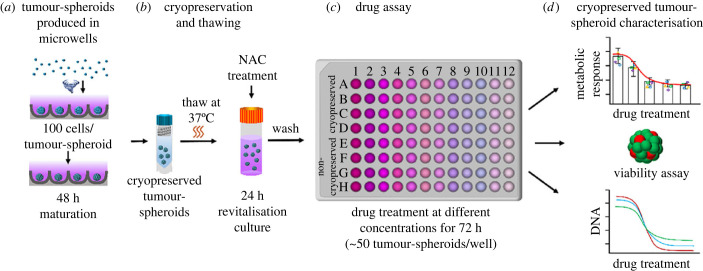
Graphical outline of the optimised experimental process. (*a*) C4-2B or MCF-7 cancer cells were seeded into microwell plates and cultured for two days. (*b*) The cancer tumour-spheroids were harvested from the microwells and cryopreserved in CryoStor CS10. Following thaw, the CS10 was removed, and tumour-spheroids were revitalised for 24 h in HG-DMEM supplemented with Happy Cell ASM,10% FBS, Pen/Strep and *N*-Acetyl-l-cysteine (NAC). (*c*) The C4-2B or MCF-7 tumour-spheroids were resuspended in fresh HG-DMEM supplemented with Happy Cell ASM,10% FBS, Pen/Strep (without NAC) and distributed into ultra-low binding 96-well plates. Approximately 50 cryopreserved or non-cryopreserved tumour-spheroids were distributed into each well in the 96-well plate. Tumour-spheroids were treated with an anti-cancer drug, Docetaxel, at 0.01–100 nM. (*d*) The drug-treated C4-2B or MCF-7 tumour-spheroids were evaluated for metabolic activity, viability and DNA content after 72 h.

**Figure 5.**
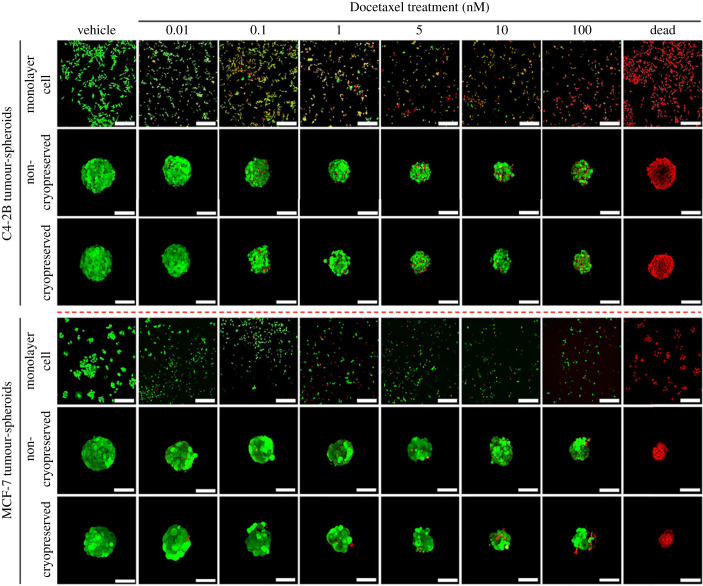
Viability of drug-treated tumour-spheroids. Tumour-spheroids were stained with calcein-AM (Live, green) and ethidium homodimer-1 (Dead, red). Column images with a ‘Dead' caption represent cells or tumour-spheroids that had been submerged in 80% ethanol for 30 min before staining, and these served as a dead control. Results are shown from three replicate experiments. Data from replicate experiments are presented in electronic supplementary material, figures S5 and S6. Scale bar = 50 µm.

The optimised manufacturing, cryopreservation, revitalisation protocol was used in the drug screening assays; see schematic in figure 4. Drug response of C4-2B and MCF-7 tumour-spheroids was assessed using a chemotherapeutic agent, Docetaxel, at various concentrations. The effect of the drug treatment was assessed by tracking tumour-spheroid viability, tumour-spheroid metabolic activity and DNA content; all measures were compared with C4-2B or MCF-7 maintained in monolayer culture. Confocal microscopy images of calcein-AM (Live, green) and ethidium homodimer-1 (Dead, red) stained tumour-spheroids and monolayer cells after drug treatment are shown in figure 5. There was notable cell death in C4-2B and MCF-7 monolayer cells in response to low concentrations (0.01 nM) of Docetaxel. The cell death increased with incrementally greater Docetaxel concentrations, and very few cells survived the highest concentration of 100 nM.

Cryopreserved and non-cryopreserved tumour-spheroids appeared similar in Live/Dead assays (figure 5). Few dead cells were observed in tumour-spheroids exposed to 5–10 nM Docetaxel. At the highest concentration, Docetaxel appeared to cause cell death at the core of the C4-2B tumour-spheroids. MCF-7 tumour-spheroid viability was compromised at Docetaxel concentrations greater than 10 nM. Comparatively, C4-2B tumour-spheroids were more sensitive to Docetaxel treatment than MCF-7 tumour-spheroids. The experiments were conducted in biological triplicate (*n* = 3) to confirm the observations. The viability images of the drug-treated cryopreserved and freeze–thawed tumour-spheroids from the other biological replicate experiments are presented in electronic supplementary material, figures S5 and S6.

**Figure 6.**
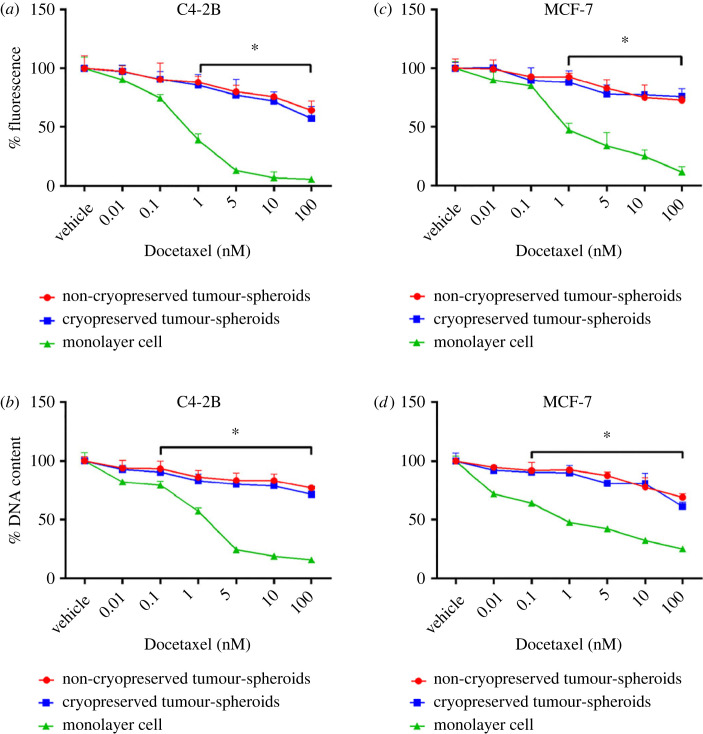
Metabolic activity and DNA content of tumour-spheroid and control monolayer cultures treated with different concentrations of Docetaxel. (*a*,*c*) The percentage of fluorescence conversion of AlamarBlue by C4-2B and MCF-7 derived cryopreserved, non-cryopreserved tumour-spheroid and monolayer cultured cells, respectively, at different concentrations of Docetaxel treatment. (*b*) DNA content of C4-2B tumour-spheroid and monolayer cultured cells after Docetaxel treatment. (*d*) The DNA content of MCF-7 tumour-spheroid and monolayer cultured cells after Docetaxel treatment. Data are presented as mean ± s.d. of four replicates normalised to vehicle control cultures. Results were obtained for three individual replicate experiments. Data from replicate experiments are presented in electronic supplementary material, figures S7 and S8. * represents *p* < 0.05, relative to monolayer cultures.

Cryopreserved and non-cryopreserved C4-2B (figure 6*a*) or MCF-7 (figure 6*c*) tumour-spheroids showed almost identical responses to Docetaxel treatments when evaluated using the AlamarBlue assay. The AlamarBlue signal declined significantly in C4-2B and MCF-7 monolayer cultures, evident from the lowest concentrations of Docetaxel (figure 6*a*,*c*). The difference between monolayer and cryopreserved or non-cryopreserved tumour-spheroid cultures was significant from 1 to 100 nM of Docetaxel (figure 6*a*,*c*). C4-2B cells, cultured in monolayers, were almost metabolically inactive with Docetaxel greater than 5 nM (figure 6*a*). MCF-7 cells showed the lowest metabolic activity at 100 nM (figure 6*c*).

When DNA was quantified in these cultures, trends similar to the metabolic assays were observed (figure 6*b*,*d*). The effect of Docetaxel was dose-dependent for both tumour-spheroids and monolayer cells. The dose-response curves were also similar for cryopreserved and non-cryopreserved tumour-spheroids. DNA content of C4-2B monolayer cells sharply declined from 0.1 to 5 nM and remained steady up to 100 nM (figure 6*b*). The declination of DNA content was gradual with the increment of Docetaxel concentration for MCF-7 monolayer cells (figure 6*d*). The percentage of DNA content for both C4-2B and MCF-7 tumour-spheroids was significantly greater than corresponding monolayer controls across the same drug concentrations (figure 6*b*,*d*). The experiments were conducted in three biological triplicates (*n* = 3). Results from biological replicate experiments are presented in electronic supplementary material, figures S7 and S8. These data were consistent with our previous report, which also studied these cell populations [17].

## 4. Discussion

Not long ago, some laboratories generated many of their own reagents, including sophisticated reagents such as antibodies or Taq polymerase. Today, most reagents are available as standardised and quality-controlled products. Deferring reagent development to supply companies provides a more effective division of labour, ultimately reduces costs, contributes to reagent consistency, and has significantly accelerated the rate of discovery in the biomedical sciences. Tumour-spheroids are growing in popularity as a culture tool, and it may be possible for tumour-spheroids, microtissues or organoids to evolve to become standardised reagents for some applications. There is already precedent for this. We cite STEMCELL Technology's sale of cryopreserved intestinal organoids [11,12], but note that ATCC also claims to offer 173 different organoid cell populations as cryopreserved products [36]. In general, cryopreserved organoids are intended to be thawed, propagated in culture, and then used in experimental studies. Our goal in this study was to push this concept to the next level, where the cryopreserved tumour-spheroids could be used directly in studies. If this could be achieved, the use of cryopreserved organoids, microtissues or tumour-spheroids could be further standardised, and it might soon be possible to directly compare results obtained in one experiment or laboratory with another, thereby overcoming some of the reproductivity challenges plaguing the field [7–9]. To optimise and test this concept, we assembled tumour-spheroids from two commonly used cancer cell lines, C4-2B prostate cancer cells and MCF-7 breast cancer cells, cryopreserved these tumour-spheroids, and then evaluated their behaviour in a drug assay.

C4-2B and MCF-7 cells were assembled into tumour-spheroids using a microwell platform, after which they were cultured for 48 h. Microwell platforms enable efficient mass production of uniformly sized tumour-spheroids [15–17,37–40]. Having uniformly sized tumour-spheroids facilitates process optimisation, and thus we reasoned that microwells were the ideal tool with which to generate input tumour-spheroids for this study. In our initial screening, we observed that tumour-spheroids assembled from 100 cells each yielded highly uniform-shaped tissues, and these tumour-spheroids performed better than tumour-spheroids assembled from 200 or 400 cells each during initial cryopreservation experiments. In these screening studies, we cryopreserved tumour-spheroids in either 90% FBS + 10% DMSO or in a commercial cryopreservation medium, CS10. Viability appeared to be superior when tumour-spheroids were cryopreserved in CS10.

Following initial cryopreservation steps, we thawed tumour-spheroids, and cultured them in HG-DMEM supplemented with 10% FBS. Visual observation of these cultures identified several problems; first, while microwell culture enabled the assembly of uniform tumour-spheroids, their subsequent culture in standard well plates led to uncontrolled and undesirable tumour-spheroids clumping, or amalgamation. Second, the clumping or disruption of clumps appeared to damage cells on the surface of tumour-spheroids, leading to death. Third, some settling and clumping of tumour-spheroids appeared likely to occur during cryopreservation in 90% FBS + 10% DMSO, but tumour-spheroids appeared to settle less (i.e. they remained partially buoyant) in CS10 cryopreservation medium.

We took the view that it would be ideal if the tumour-spheroids could be maintained in a suspension, thereby minimising clumping, and maintaining the useful discrete uniform tumour-spheroid product generated in the microwell platform. Based on these observations, we used CS10 cryopreservation medium in subsequent experimentation to minimise tumour-spheroid aggregation during the cryopreservation process. Happy Cell ASM, is a novel neutral buoyancy medium, that, according to the manufacturer, is a ‘unique, low viscosity, low density, cell culture reagent that permanently suspends cells'. Based on these promising properties, we trialled thawing tumour-spheroids into and culturing tumour-spheroids in Happy Cell ASM medium. These two modifications contributed to better maintenance of discrete tumour-spheroids with high viability.

In addition to tumour-spheroid assessment with LIVE/DEAD viability dye, we characterised tumour-spheroid metabolism using the AlamarBlue assay. Despite high viability, tumour-spheroid metabolic activity was significantly reduced following cryopreservation. Previous studies have reported impaired cellular aerobic respiration and mitochondrial function following cryopreservation [41–44]. A single freeze–thaw cycle is sufficient for uncoupling mitochondria and disrupting mitochondrial membranes [41]. Alteration of mitochondrial membrane conformation and membrane potential stimulate the generation of ROS [45], responsible for damaging the mitochondrial NADPH dehydrogenase [46,47]. AlamarBlue is reduced by mitochondrial NADH dehydrogenase [29], and thus the dampened AlamarBlue signal following cryopreservation could indicate ROS-mediated mitochondrial damage. ROS scavengers and apoptotic inhibitors have been used in cryopreservation processes to improve cell viability [48,49], and one study indicated that NAC medium supplementation post-thaw improved metabolic performance [50]. NAC is an ROS inhibitor capable of scavenging H_2_O_2_ and **^·^**OH, as well as contributing to glutathione replenishment and protecting sulfhydryl groups on the mitochondrial membrane [50]. We postulated that the addition of NAC to the medium during thawing and during a recovery period following thawing might restore tumour-spheroid metabolic function. We tested this concept and developed a post-thaw recovery protocol where tumour-spheroids were cultured in Happy Cell ASM, supplemented with 10% FBS and 10 µM NAC. When the medium was supplemented with 10 µM NAC, viability was maintained, and respiration, as measured by the AlamarBlue assay, was similar for fresh and cryopreserved tumour-spheroids. Increasing the NAC concentration appeared to further elevate metabolic activity as measured by the AlamarBlue assay, until 1 mM, after which point greater NAC appeared to be toxic. C4-2B cells and MCF-7 cells appeared to behave slightly differently, suggesting that NAC concentration may need to be optimised for individual cell types.

In our study, thawed tumour-spheroids were exposed to NAC for 24 h in a ‘revitalisation' step prior to their use in the drug assays. NAC was depleted by washing the tumour-spheroids before transferring them into the drug assay plate. Thus, our study did not evaluate how the combination of NAC and drug might have influenced the drug assay outcomes. Data in electronic supplementary material, figures S2 and S3 show the significant increase in metabolic activity in non-frozen tumour-spheroids exposed to 10 µM NAC. Noting the caveat that we have not tested this, we presume that having NAC in the culture medium used in the drug assay may confound assays outcomes; possibly elevating respiratory activity in cells that are dying and where you might expect to observe a declining respiratory signal. For this reason, it is necessary to have a staged process where thawed tumour-spheroids are treated with NAC, the NAC then eliminated, and then the tumour-spheroids used in drug assays. Critically, it does appear that cell respiration in tumour-spheroids is compromised following cryopreservation. A more refined process would be one that reduces the impact of cryopreservation, perhaps allowing the tumour-spheroids to be used directly, or earlier following thaw, in drug assays. Agilent Seahorse XF Technology allows precise quantification of cell metabolic activity, and a rational next step might be to characterise metabolic activity prior to and following cryopreservation, and to use the Agilent Seahorse XF to assess the benefits of various cryopreservation medium supplements to either protect against damage or to more rapidly restore metabolic function. Likely, it will be possible to shorten or eliminate the ‘revitalisation’ step currently integrated into our protocol.

The cryopreserved tumour-spheroids were assessed for drug response using the anti-cancer drug, Docetaxel. Docetaxel binds and stabilises microtubules, leading to cell cycle arrest and apoptosis [51]. Treatment with the drug diminished metabolic output and DNA content and caused significant cell death in C4-2B and MCF-7 monolayer cultures. By contrast, and similar to previous reports [16,17], 3D tumour-spheroids were less sensitive to drug treatment [52,53]. Drug response of non-cryopreserved and cryopreserved tumour-spheroids was nearly identical, demonstrating the effectiveness of the iterative optimisation and the potential utility of this process. A limitation of the describe study is that the method was only tested using cell lines, which are easily manipulated but do not mimic patient drug response as well as primary cancer cells [54]. A logical next step would be to optimise our methodology to work with cancer cells enriched from patient-derived xenografts. Dr Kathleen Kelly and colleagues previously described the development of a method for culturing and cryopreserving organoids derived from prostate cancer PDXs [54]. Their data suggest that extension of our tumour-spheroids cryopreservation methodology to include use of cells enriched from patient-derived xenografts may be feasible. Their PDX lines represent a broad range of different stages/types of prostate cancer [54], and if it were possible to establish tumour-spheroids ‘ready to go' into drug assays, this would likely have significant utility, and would represent a logical next step in technology development.

## 5. Conclusion

Herein, we optimised a process for manufacturing cancer tumour-spheroids in microwells, their cryopreservation in CS10 medium, recovery in Happy Cell ASM, supplemented with 10% FBS and 10 µM NAC, then distribution of tumour-spheroids into 96-well plates again in Happy Cell ASM, supplemented with 10% FBS to study drug response. The success of this process revolves around three critical considerations: (i) Optimisation is facilitated by using a uniform tumour-spheroid product, and we recommend a microwell platform. (ii) Tumour-spheroid aggregation should be minimised during freezing, manipulation and during culture. This can be achieved in part through buoyancy conferred by CS10 cryopreservation medium and by Happy Cell ASM cell culture medium. (iii) Cell respiration is impaired following cryopreservation, but this can be rescued by including a recovery culture where the medium is supplemented with NAC. Limitations to the described process include the necessity of a recovery step, and the fact that we have not yet adapted the process to more challenging primary cell populations. Nevertheless, this process does provide an initial road map that may guide future efforts to develop tumour-spheroid banks, that contain tumour-spheroids ready-to-go as standardised inputs into experiments conducted around the world.

## Data Availability

We have uploaded the data as a supplementary file [55].
